# Immune checkpoint therapy in liver cancer

**DOI:** 10.1186/s13046-018-0777-4

**Published:** 2018-05-29

**Authors:** Feng Xu, Tianqiang Jin, Yuwen Zhu, Chaoliu Dai

**Affiliations:** 1 Department of Hepatobiliary and Splenic Surgery, Shengjing Hospital affiliated to China Medical University, Shenyang, 110004 Liaoning China; 2 Department of Surgery, University of Colorado Anschutz Medical Campus, RC1-North Building, P18-8116, Aurora, CO 80045 USA

**Keywords:** Immune checkpoint, Hepatocellular carcinoma, Cholangiocarcinoma, Immunotherapy, Epigenetics

## Abstract

Immune checkpoints include stimulatory and inhibitory checkpoint molecules. In recent years, inhibitory checkpoints, including cytotoxic T lymphocyte–associated antigen 4 (CTLA-4), programmed cell death protein-1 (PD-1), and programmed cell death ligand 1 (PD-L1), have been identified to suppress anti-tumor immune responses in solid tumors. Novel drugs targeting immune checkpoints have succeeded in cancer treatment. Specific PD-1 blockades were approved for treatment of melanoma in 2014 and for treatment of non-small-cell lung cancer in 2015 in the United States, European Union, and Japan. Preclinical and clinical studies show immune checkpoint therapy provides survival benefit for greater numbers of patients with liver cancer, including hepatocellular carcinoma and cholangiocarcinoma, two main primary liver cancers. The combination of anti-PD-1/PD-L1 with anti-CTLA-4 antibodies is being evaluated in phase 1, 2 or 3 trials, and the results suggest that an anti-PD-1 antibody combined with locoregional therapy or other molecular targeted agents is an effective treatment strategy for HCC. In addition, studies on activating co-stimulatory receptors to enhance anti-tumor immune responses have increased our understanding regarding this immunotherapy in liver cancer. Epigenetic modulations of checkpoints for improving the tumor microenvironment also expand our knowledge of potential therapeutic targets in improving the tumor microenvironment and restoring immune recognition and immunogenicity. In this review, we summarize current knowledge and recent developments in immune checkpoint-based therapies for the treatment of hepatocellular carcinoma and cholangiocarcinoma and attempt to clarify the mechanisms underlying its effects.

## Background

Globally, primary liver cancer accounts for 6% of all cancers and 9% of all death from cancer. It is the sixth most common cancer and the second leading cause of cancer death. The important primary liver cancers include hepatocellular carcinoma (HCC), accounting for approximately 75%, and cholangiocarcinoma, accounting for approximately 6%. Although either surgical resection or liver transplant can be used for the treatment of liver cancer, limitations are caused by high recurrence rates after resection and low-ratio eligibility for surgery and transplant because this cancer is often detected at a late stage [1, 2]. In the tumor microenvironment, cancer cells and host immune responses interact to promote or inhibit the pathologic progression of cancer. The immune system can identify cancer cells, and mobilizing the immune response is able to eliminate cancer [3]. Immunotherapy has emerged as a promising therapy and is being investigated in various tumors including liver cancer [4]. Emerging evidence supports that the blockade of immune checkpoints is among the most promising approaches in cancer immunotherapy [4–6].

**Fig. 1:**
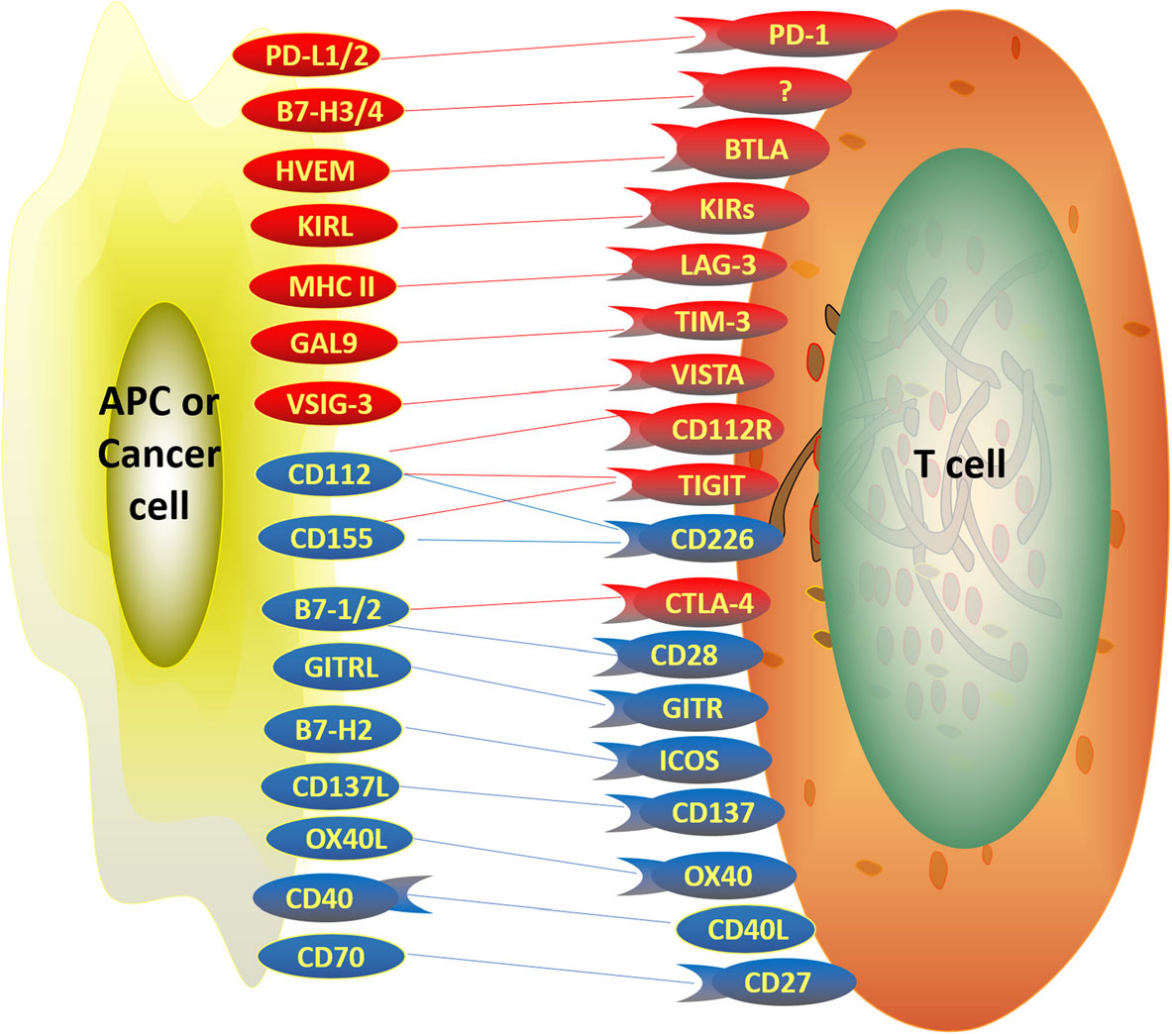
Illustration of stimulatory and inhibitory immune checkpoints between T-cells, APCs, and cancer cells. Blockade of inhibitory immune checkpoints can positively regulate T-cell activation and prevent immune escape of cancer cells within the tumor microenvironment. Activation of stimulatory immune check points can augment the effect of immune checkpoint inhibitors in cancer therapeutics. Red, inhibitory immune checkpoints; blue, stimulatory immune checkpoints

The activity of the immune system is mostly regulated by immune cells called T cells. In the tumor microenvironment, T cells can recognize tumor antigens, which are presented to T cell receptors by antigen-presenting cells (APCs). Besides signal via T cell receptors, T cell response is fine-tuned by a group of cell surface molecules, named immune checkpoints. They can be either stimulatory or inhibitory, and participate in various stages of T cell response (Fig. 1) [6–11]. Many cancers are able to evade the immune system, mainly by overexpressing inhibitory ligands to damp T cell attack. As a result, fewer, and damaged T cells were found in patients with HCC, which contributed to the progression of this cancer [12].

Recently, in vitro and in vivo results show histone deacetylase inhibitors (HDACi) and DNA methyltransferase inhibitors (DNMTi), two important epigenetic drugs, can up-regulate expression of inhibitory immune checkpoints in either immune or cancer cells [13–15]. Epigenetic modifiers function importantly in priming and enhancing the therapeutic effect of the host immune system on cancer [14, 15]. The purpose of this review is to give a brief overview of the role for immune checkpoints related to liver cancer progression. It also provides new insights into the epigenetic mechanism in checkpoint immunotherapy and checkpoint blocking – based therapeutic approaches for treatment of liver cancer.

## Immune checkpoints and hepatocellular carcinoma

The most ex vivo studied and clinically relevant checkpoint proteins are CTLA-4, PD-1, and PD-L1 (Tables 1 and 2). The expression of inhibitory immune checkpoints can be dysregulated in a tumor microenvironment, which can lead to improvement of T cell-mediated immune response through cancer immunotherapy [16]. The PD-1 pathway is found to suppress T cell activation mainly within peripheral tissues at the later phase, whereas the CTLA-4 pathways are involved in regulation of T cell-mediated immune responses primarily in lymph nodes at the priming phase [17].

**Table 1:** Immune checkpoints expression in liver cancers

Cancer type	Number	TNM Stage (I + II / III + IV)	Tumor differentiation (I + II / III + IV)	Tumor size (cm)	Immune checkpoints	Cellular expression	Year	Reference
Human HCC	217 (tumor samples)	Operable, resected	101 (46%)/ 116 (53%)	7.26 (1.0–2.5)	PD-L1/PD-1	neoplastic and inflammatory cells	2016	[24]
Human HCC	176	97/52	112/64	5.3 (PD-L1 _low_)/4.9 (PD-L1 _high_)	PD-L1	CD68+ macrophages	2016	[27]
Human HCC	90	Operable, resected	73/17	4.2 (1.3–15)	PD-L1	peritumoral hepatocytes	2017	[25]
Human HCC	294	59/87	140/6	110(<5) /36(≥5)	PD-L1/PD-1 and CTLA-4	tumor infiltrating	2017	[26]
Human HCC	69	35/34	50/19	7/21(Tim-3 low)/17/24 (Tim-3 high)	Tim-3	CD14+ monocytes	2015	[28]
Human HCC	171	100/71	NR	98/73	PD-1 and Tim-3	neoplastic and inflammatory cells	2016	[29]
Human ICC	31	9/22	13/18	20 (<5) / 11 (>5)	PD-L1 and PD-1	neoplastic and inflammatory cells	2009	[38]
Human ICC	27	16/11	19/8	NR	PD-L1	ICC cells	2016	[36]

*HCC* hepatocellular carcinoma, *ICC* Intrahepatic cholangiocarcinoma, *NR* not reported

**Table 2:** Pre-clinical studies with immune checkpoints in therapy of liver cancers

Cancer type	Number	TNM Stage (I + II / III + IV)	Tumor differentiation (I + II / III + IV)	Tumor size (cm)	Immune checkpoints	Therapy	Target cells	Year	Reference
Human HCC	71	57 / 14	58 / 13	36 (≤5) / 35 (>5)	PD-L1 and PD-1	PD-L1and PD-1 antibodies	Kupffer cells and CD8^+^ T cells	2009	[23]
Human HCC	NR	NR	NR	NR	PD-L1	Specific shRNA for PD-L1 and DNMT1	HCC cell lines	2017	[57]
Human HCC	31	22/9	21/10	9(≤5) /22(>5)	CTLA-4	CTLA-4 antibodies	Tumor-Associated Antigen-Specific T Cells	2011	[16]
Mice HCC	NR	NR	NR	NR	CTLA-4	CTLA-4 antibodies	Regulatory T cells	2017	[21]
Human HCC	59	54 / 4 unknown, *n* = 1	NR	NR	LAG3, PD-1, Tim3 and CTLA4	Blocking antibodies to LAG3, PD-1, TIM3 or CTLA4	tumor-infiltrating T cells	2017	[30]
Human HCC	21	8/13	NR	NR	GITR	GITR ligand	tumor-infiltrating Tregs	2013	[33]

*HCC* hepatocellular carcinoma, *ICC* Intrahepatic cholangiocarcinoma, *NR* not reported

### CTLA-4

CTLA-4 is a CD28 homolog and primarily located in intracellular compartments in resting naive T cells. CTLA-4 inhibits T cell response by directly delivering an inhibitory signal to T cell, and interfering with the binding between B7 and CD28 [18]. In 31 HCC patients, it was found the addition of anti-CTLA-4 antibody resulted in an increase in the frequency of tumor-associated antigens (TAA)-specific cytotoxic T cells in 60% of HCC patients, accompanied with enhanced antitumor effect of tumor-specific T cells [19]. In addition, CTLA-4 is shown to be important for regulatory T cell (Treg) function. Tregs control functions of the effector T cells, and thus crucially maintain peripheral tolerance [20]. Unlike effector T cells, Tregs constitutively express CTLA-4 to exert their immune suppression [21, 22]. Treg-specific CTLA-4 deficiency was shown to affect in vivo Treg suppressive function and promote tumor immunity [21, 22]. In a rat liver transplantation model with tumor recurrence, hepatic expressions of CTLA-4, TGF-β and PD-L1 were increased in the tumor tissues from small-for-size liver graft group compared to whole graft group. The results suggested that up-regulation of CTLA-4 may mediate the mobilization of Tregs by small-for-size graft injury, contributing to HCC recurrence after liver transplantation [23]. HCC-derived Tregs down-regulated CD80/86 expression on splenic DCs in a CTLA-4 dependent manner, and inhibition of CTLA-4 could prevent the Treg-mediated suppression in anti-tumor immune responses [24]. Thus, CTLA-4 could not only enhance the antitumor effect of effector T cells but also maintain self-tolerance and the suppressive function of Tregs in liver cancer immunity.

### PD-1/PD-L1

PD-L1 is the main ligand for PD-1, which is crucial for tumor immunity. In addition, PD-L1 also interacts with B7-1 to inhibit T cell immunity, and the role of this interaction in cancer immunity is still unclear [25]. Binding of PD-L1 to its receptor can suppress T cell migration, proliferation, and secretion of cytotoxic mediators, and thus blocks the “cancer immunity cycle” [26]. In the HCC tumor microenvironment, PD-L1 expression is mainly expressed in Kupffer cells but is slightly expressed on other APCs or HCC tumor cells [27]. CD8+ T cells and Kupffer cells in human HCC tumor tissues expressed high levels of PD-1 and PD-L1, respectively. PD-L1+ Kupffer cells interact with PD-1 + CD8+ T cells and contribute to dysfunction of effector T cells in HCC. Elevated PD-L1 expression in HCC is indeed associated with poorer prognosis in HCC patients [27]. In 217 HCCs, PD-L1 was expressed by both neoplastic and intra-tumoral inflammatory cells, which are related to tumor aggressiveness. It also suggests that the PD-L1/PD-1 immune checkpoint could be targeted in the treatment of particular HCC variants [28]. More recently, 90 HCC patients with PD-L1 expression in peritumoral hepatocytes were demonstrated to have a significantly higher risk of cancer recurrence or metastasis and cancer-related death [29]. Immunohistochemistry data in 294 HCC tissue samples showed PD-1 and PD-L1 expression was significantly related to high CD8+ tumor-infiltrating lymphocytes (TILs). Only high Edmondson–Steiner grade was markedly related to high PD-1 expression. High PD-L1 expression was demonstrated as an independent poor prognostic factor for disease-free survival in the high CD8+ TILs group. Further, combined high expression of PD-L1 and CD8+ TIL is an important prognostic factor related to the immune checkpoint pathway in HCC. Also, this result would be helpful in evaluating the applicable group of PD-1/PD-L1 blocking agent for HCC patients [30]. PD-L1 expression was significantly increased in tumors with a high number of tumor-infiltrating lymphocytes (*ρ* = 0.533, *p* < 0.001). High PD-L1 expression was associated with significantly shorter overall survival [31]. These clinic data further support that PD-L1 is an important mediator in the progression and an important target in the anti-tumor therapy for liver cancer.

### Other inhibitory checkpoints

Several other inhibitory receptors, including T-cell immunoglobulin- and mucin-domain-containing molecule-3 (Tim-3) and LAG-3, are also upregulated on TAA-specific CD8+ T-cells in various cancer types, and are also involved in progression of liver cancer. Tim-3 is strongly expressed on CD4+ and CD8+ T-cells obtained from HCC lesions in contrast to the surrounding liver tissue. Tim-3 is expressed on tumor-associated macrophages (TAM), which contributes to HCC growth [32]. Intriguingly, a high number of Tim3+ tumor infiltrating cells and Tim3+ TAM in HCC lesions are associated with a poor prognosis [33]. In 171 patients with hepatitis B virus (HBV)-related HCC, both PD-1 and Tim-3 expressions in liver infiltrating lymphocytes were significantly high in tumor tissues compared to tumor adjacent tissues. The up-regulation of PD-1 and Tim-3 were related to higher tumor grades [33]. There is a significant positive intercorrelation between the levels of PD-1 and Tim-3 expression in tumor tissues and tumor adjacent tissues. The expressions of PD-1 and Tim-3 in tumor tissues and tumor adjacent tissues were significantly associated with PD-1 and Tim-3 polymorphisms, with genotype AA of PD-1 rs10204525 and genotypes GT + TT of Tim-3 rs10053538 respectively [33]. LAG-3 is another important inhibitory immune check point and exerts synergistic effects with PD-1/PD-L1 on T cell activation in the tumor microenvironment. In HCC-vaccine-immunized mice, STAT3-blocked HCC vaccine downregulated expression of PD-1, TIGIT, and LAG-3, which could prevent cancer-induced dysfunction of CD8+ T and natural killer cells [34]. Recently, expression of LAG3 was found to be significantly higher on tumor-associated antigen (TAA)-specific CD8+ tumor-infiltrating T helper cells and CD8+ cytotoxic T cells in tumors than those in tumor-free liver tissues and blood of HCC patients [35]. Interestingly, blocking LAG-3 increased ex vivo proliferation of CD4+ and CD8+ TIL and effector cytokine production. Combination of LAG-3 blocking antibody with PD-L1 blockade further augmented TIL responses to polyclonal stimuli and TAA [35]. This suggests that LAG-3 plays an important role in T-cell suppression in the HCC microenvironment and might be a promising immunotherapeutic target for HCC. Further clinical trials about Tim-3, Lag-3 or TIGIT blockers should be performed in liver cancer treatment.

### Co-stimulatory immune checkpoints

The best characterized co-stimulatory ligands that have been investigated in hepatocellular carcinoma are B7-1 and B7-2. These two important immune checkpoints are mainly expressed on professional antigen-presenting cells. B7-1 and B7-2 can bind to both CD28 and CTLA-4, and thus regulate T cell activation via selective interacting with either CD28 or CTLA-4 [36]. Expression of costimulatory molecules, including B7-1 and B7-2, have been found to be down-regulated in HCC cells [37]. This down-regulation may lead to suppression of activation of effector T-cells mediated by B7/CD28. The glucocorticoid-induced tumor necrosis factor receptor (GITR) and the inducible T-cell co-stimulator (ICOS) are co-stimulatory checkpoints and regulate the immunosuppressive Tregs function. Importantly, GITR and ICOS are up-regulated in Tregs infiltrating HCC and may function as potential targets for immunotherapeutic interventions for antitumor therapy [38].

## Immune checkpoints and cholangiocarcinoma

Intrahepatic cholangiocarcinoma (ICC) represents the second most common primary liver malignancy, accounting for 10–20% of all primary liver cancers [39]. Although ICC is traditionally viewed as a rare cancer, its incidence has been steadily rising, with recent reports showing the incidence of ICC in the USA has increased from 0.44 to 1.18 cases/100,000 over the past three decades [40]. The prognosis for ICC continues to be poor, with surgery as the only definitive option for cure. Median survival rate is low because most patients are not eligible for curative resection. As such, there is an increasing need for the development of novel adjuvant therapies for patients with ICC.

### PD-1/PD-L1

In contrast to HCC, immunotherapy in cholangiocarcinoma has been limited and mostly ineffective [41]. However, a high frequency of tumor-infiltrating lymphocytes and PD-L1 expression suggest that checkpoint inhibition may prove effective [42]. Expression of PD-L1 was found both in tumor-associated macrophages and in the tumor front. Patients with tumors exhibiting PD-L1 expression around the tumor front had a lower overall survival than tumor front-positive patients [43]. In 31 surgically resected ICC samples from Asian patients, PD-L1 expression was significantly higher in tumor tissue than that in adjacent tissue [44]. High levels of PD-L1 expression were also found in Western patients with ICC, which resulted in tumor poor differentiation, higher malignant tumor stage and higher levels of apoptotic CD8+ TILs, and therefore led to lower chance of survival [42]. More recently, in occupational cholangiocarcinoma, PD-L1 expression was found in biliary intraepithelial neoplasia and intraductal papillary neoplasm. Cholangiocarcinoma cells expressed PD-L1 in a low number of cases of occupational cholangiocarcinoma, while carcinoma cells expressed PD-L1 in all cases. Moreover, PD-L1 and PD-1 were also expressed in tumor-associated macrophages and tumor-infiltrating T cells expressed. The number of PD-L1-positive mononuclear cells, PD-1-positive lymphocytes, and CD8-positive lymphocytes infiltrating within the tumor was markedly high in occupational cholangiocarcinoma. Immunostaining with mAbs detected human leukocyte antigens (HLA) class I defects in 60% of ICC tumors and PD-L1 expression in 30%. Patients bearing tumors with HLA class I defects and PD-L1 expression had a significantly reduced survival rate. The results suggested PD-L1 up-regulation mediates immune escape in cholangiocarcinoma and could be potential biomarker of response to anti-PD-1/PDL1 immunotherapy [45]. The role of other immune checkpoints for cholangiocarcinoma is still not well established.

## Epigenetic mechanism in checkpoint immunotherapy

In cancer, two important epigenetic mechanisms include hypermethylation, which is mediated by DNMTs, and histone deacetylation, which is mediated by HDACs. Epigenetic dysregulation is a crucial mechanism underlying the progression of cancer [46–49]. Some epigenetic regulators can act negatively and positively in immune responses and lead to immune evasion [50], which provides a novel mechanism in immune checkpoint therapy for treatment of cancers.

Recently, epigenetic modifications of the key immune checkpoints including PD-1, PD-L1, and CTLA-4 were analyzed in non-small cell lung cancer tissues from 39 patients [51]. It was shown that CTLA-4 and PD-1, but not PD-L1, are hypomethylated in human lung tumors. This hypomethylation also led to increased expression of these two genes as shown by transcriptome analysis [51]. In a phase 2 trial, hypomethylating agents such as vorinostat and azacitidine upregulated mRNA expression of PD-L1, PD-L2, PD-1 and CTLA-4 in 61 patients with acute myeloid leukemia [52]. More recently, profiling DNA methylation in peripheral blood mononuclear cells and T cells from HCC patients show that a broad signature of DNA methylation intensifies with progression of HCC [53]. Importantly, HCC DNA methylation is highly enriched in immune function-related gene PD-1 [53]. Interestingly, Liu et al. found highly upregulated DNA methyltransferase 1 (DNMT1) is positively correlated with PD-L1 overexpression in sorafenib-resistant HCC cells. PD-L1 further induced DNMT1-dependent DNA hypomethylation and restored the expression of methylation-silenced Cadherin 1, a metastasis suppressor in HCC [54].

Accumulating evidence also shows histone deacetylation regulates immune checkpoint expression and plays an important role in cancer progression. HDAC is have been shown to sensitize cancer cells to immune checkpoint therapy by upregulating the immune checkpoints CTLA-4, PD-1, PD-L1, and PD-L2 on tumor cells and TILs [55]. For example, inhibition of the class I HDAC1, HDAC2 and/or HDAC3 led to acetylation of the PD-L1 and PD-L2 promotors, which augmented up-regulation of PD-L1/L2 protein and RNA transcription in melanoma patients, in melanoma cell lines and in a syngeneic mouse model of melanoma [56]. Interestingly, Lienlaf et al. [57] found HDAC6i (ACY-241) reduced PD-L1 production and increased co-stimulatory checkpoint (CD28) levels, and thus suppressed tumor growth in vivo. In the WM164 HDAC6KD cells, the expression of PD-L2, B7-H4 and TRAIL-R1 were largely diminished, while B7-H3, Galectin-9 and TRAIL-R2 were moderately decreased. In breast cancer cells, CD137, a co-stimulatory checkpoint, was found to be up-regulated by HDACi (SAHA) treatment [58]. Therefore, inhibitory and co-stimulatory checkpoints can be up-regulated or down-regulated by different HDAC isoforms in different tumor types. To date, the immune modulatory activity of HDAC inhibitors on tumor-specific immunity including immune checkpoints has not been well demonstrated or characterized in HCC.

Recent evidence suggests that noncoding RNAs, such as microRNAs (miRNAs) and long noncoding RNAs (lncRNAs), may also have direct epigenetic functions by recruiting specific protein complexes to genomic DNA, and specifically to some promoters modulating the expression of the corresponding genes. MiRNAs and lncRNAs play important roles in regulating expression of immune checkpoints in various tumors [59]. In human malignant pleural mesothelioma, the levels of miR-15b, miR-16, miR-193a-3p, miR-195, and miR-200c were significantly lower in the immune checkpoint PD-L1-positive samples. Likewise, PD-L1 and miR-138-5p levels were inversely correlated in human colorectal cancer tumors, and miR-138-5p inhibited PD-L1 expression in tumor models in vivo [60]. In lung cancer, it was demonstrated that the p53/miR-34/PD-L1 and miR-200/ZEB1/PD-L1 axis are novel mechanisms in tumor immune evasion [61, 62]. Moreover, it is recently demonstrated that transfection of human CD4+ T cells with miR-138 suppressed expression of CTLA-4, PD-1, and Foxp3 in glioma preclinical models [63]. Whether the association between miRNA expression and immune checkpoint levels in tumors can be translated into a predictive marker of checkpoint inhibitor therapy in liver cancer requires further investigation. Interactions among three kinds of RNAs were revealed in the ‘lncRNA-miRNA-mRNA’ competing endogenous RNA network. Several biomarkers were identified for diagnosis of diabetic pancreatic cancer, such as lncRNAs (HOTAIR, CECR7 and UCA1), hsa-miR-214, hsa-miR-429, CCDC33 and CTLA-4. Notably, interactions of ‘CECR7-hsa-miR-429-CTLA4’ were highlighted in the endogenous RNA network, which is very important in enhancing the progression of pancreatic cancer [64]. Some miRNAs and lncRNAs might be involved in the “cancer immunity cycle” regulated by immune checkpoints such as CTLA-4 and PD-L1-PD-1 and could be the subject of future investigations in liver cancer.

Taken together, a wave of translational research highlights the mechanistic and functional link between epigenetic regulation and immune checkpoints in the development and progression of primary tumors including liver cancer.

## Checkpoint-blocking based therapeutic approaches

Over the last decade, there has been significant progress in our understanding of the immune system which has led to development of numerous immune checkpoints blockades that have altered the management and prognosis in some cancers including liver cancer (Table 2). As more such drugs are developed, we will have multiple additional options and indications for these inhibitors in the near future. Among these pathways, the PD-1/PD-L1 and the B7-1/B7-2/CTLA-4 have been identified as clinically available inhibitors.

These immune checkpoint drugs such as nivolumab, pembrolizumab, and ipilimumab have already been FDA approved in non-small cell lung cancer, renal cell carcinoma, melanoma, Hodgkin lymphoma, and urothelial bladder cancer [65]. Trials investigating immune checkpoint blockades in HCC and cholangiocarcinoma are in progress and early signals of efficacy have recently been reported (Table 3). Encouraging clinical outcomes were reported from an ongoing phase I/II trial of the anti-PD-1 antibody nivolumab at the 2015 American Society of Clinical Oncology (ASCO) Annual Meeting held in Chicago [66]. Waterfall plots showed that the tumor size decreased to some extent in all cohorts including uninfected, HBV-infected, and hepatitis C virus-infected HCC patients. It was significant and stable in the response to the treatment of nivolumab in HCC patients. In another recent ongoing trial of nivolumab treatment in HCC patients, nivolumab showed a manageable safety profile, including acceptable tolerability. The objective response rate was 20% (95% CI 15–26) in patients treated with nivolumab 3 mg/kg in the dose-expansion phase and 15% (95% CI 6–28) in the dose-escalation phase [67]. Early data from the biliary tract cohort of Keynote-028 reported an objective response rate of 17% and a further 17% achieved stable disease in PD-L1 positive pretreated advanced cholangiocarcinoma [68].

**Table 3:** Clinical trials with immune checkpoints therapy in liver cancers

Cancer type	Number	Study arms	Stage	Status	Design	Primary outcome	Estimated completion	Trial NCT
HCC	35	Nivolumab (anti PD-1 Ab) + LRT (Yttrium 90Y glass microspheres)	Phase 1	Recruiting	Single Group Assignment	July 2019	July 2019	NCT02837029
HCC	154	PDR001 (anti PD-1 Ab) + NIS793 (anti TGF-b Ab)	Phase 1	Recruiting	Non-Randomized	January 12, 2020	January 12, 2020	NCT02947165
HCC	114	Durvalumab (anti PD-1L Ab) + ramucirumab (anti-VEGF-R2 Ab)	Phase 1	Recruiting	Non-Randomized	March 2018	September 2018	NCT02572687
HCC	51	Durvalumab (anti PD-1 L Ab) + AZD4635	Phase 1	Recruiting	Non-Randomized	November 9, 2017	November 9, 2017	NCT02740985
HCC	61	Tremelimumab (anti CTLA-4 Ab)	Phase 1	Active, not recruiting	Non-Randomized	December 31, 2017	December 31, 2018	NCT01853618
Liver cancer	60	Ipilimumab (anti CTLA-4 Ab) + MGN1703 (Toll-like receptor agonist)	Phase 1	Recruiting	Non-Randomized	May 2019	May 2019	NCT02668770
HCC	120	Ipilimumab (anti CTLA-4 Ab) + stereotactic body radiation	Phase 1	Recruiting	Randomized	August 2019	August 2019	NCT02239900
HCC	75	Nivolumab (anti PD-1 Ab) + galunisertib (TGF-b inhibitor)	Phase 1/2	Recruiting	Non-Randomized	April 2018	March 2019	NCT02423343
HCC	620	Nivolumab (anti PD-1 Ab) + ipilimumab (anti CTLA-4 Ab)	Phase 1/2	Recruiting	Non-Randomized	July 22, 2018	July 9, 2019	NCT01658878
HCC	108	PDR001 (anti PD-1 Ab) + INC280 (c-Met inhibitor)	Phase 1/2	Recruiting	Non-Randomized	December 24, 2018	December 24, 2018	NCT02795429
HCC	50	Prembrolizumab (anti PD-1 Ab) + dendritic cells, cytokine-induced killer cells	Phase 1/2	Recruiting	Single Group Assignment	September 2019	October 2019	NCT02886897
HCC	15	Prembrolizumab (anti PD-1 Ab)	Phase 1/2	Recruiting	Single Group Assignment	December 2019	December 2019	NCT02940496
HCC	50	Nivolumab (anti PD-1 Ab) + CC-122 (immunostimulatory pathway modifier)	Phase 1/2	Recruiting	Single Group Assignment	June 23, 2020	June 23, 2020	NCT02859324
HCC	90	Durvalumab (anti PD-1 L Ab), Tremelimumab (anti CTLA-4 Ab) + LRT	Phase 1/2	Recruiting	Non-Randomized	April 30, 2020	April 30, 2021	NCT02821754
HCC	620	Nivolumab (anti PD-1 Ab), Nivolumab + Ipilimumab, Nivolumab + cabozantinib, Nivolumab + Ipilimumab + cabozantinib	Phase 1/2	Recruiting	Non- Randomized	September 4, 2018	July 9, 2019	NCT01658878
HCC	28	Pembrolizumab (Keytruda) (anti PD-1 Ab)	Phase 2	Recruiting	Single Group Assignment	April 2018	April 2019	NCT02658019
HCC	440	Durvalumab (anti PD-1 L Ab) + Tremelimumab (anti CTLA-4 Ab)	Phase 2	Recruiting	Randomized	March 20, 2020	March 20, 2020	NCT02519348
HCC	726	Nivolumab (anti PD-1 Ab)	Phase 3	Recruiting	Randomized	October 1, 2018	June 22, 2019	NCT02576509
HCC	408	Prembrolizumab (anti PD-1 Ab)	Phase 3	Active, not recruiting	Randomized	February 1, 2019	February 1, 2019	NCT02702401
HCC	1200	Durvalumab (anti PD-1 L Ab) + tremelimumab (anti CTLA-4 Ab)	Phase 3	Not yet recruiting	Randomized	February 27, 2020	March 29, 2021	NCT03298451

Immunotherapy is promising for HCC and cholangiocarcinoma. However, even for those patients who respond to the single agent immunotherapy, combinational therapy may be more potent and lead to more durable response. At the 2016 ASCO meeting, an ongoing phase I trial showed trans catheter arterial chemoembolization. Radiofrequency, or cryoablation induced a peripheral immune response which may enhance the effect of anti-CTLA-4 treatment. This combination is safe and leads to the accumulation of intratumoral CD8+ T cells and activation of T cells in peripheral blood in responding patients. Encouraging clinical activity was seen with objective confirmed responses and a PFS of 5.7 months (NCT01853618) [65]. Another pilot study for the combined effect of immune checkpoint blocking and ablative therapies has been initiated in patients with advanced liver cancer (NCT02821754). Chemotherapy such as cisplatin can reduce PD-L2 expression on tumor cells [69, 70]. Both these studies show that chemotherapy can enhance antitumor immunity and thus may combine and augment immune checkpoint therapy for treatment of liver cancer.

As previously discussed, epigenetic modulators enhance cell surface expression of immune checkpoints. Several studies provided evidence to support increased expression of checkpoint inhibitors on tumor cells following epigenetic treatment, which enhances responses to immune checkpoint therapy [56, 71]. Recently, the role of HDACi and histone methyltransferases in tumor immunity and cancer therapy has been investigated. In melanoma-bearing mice, HDACi upregulated expression of PD-L1 and PD-L2 through increased histone acetylation. Further, combination of HDACi and PD-1 blockade led to higher efficiency in slowing tumor progression and improving survival rate than single agent therapy [56]. 3-Deazaneplanocin A and 5-aza-2′deoxycytidine, two important DNMTi, enhanced the therapeutic efficacy of PD-L1 blockade in reducing tumor volume, increasing tumor infiltrating CD8+ T cells and Th1-type chemokine expression in ovarian cancer in C57/BL6 mice [72]. Chiappinelli et al. demonstrated that 5-azacytidine, sensitized tumors to anti-CTLA-4 immune checkpoint therapy compared to 5-azacytidine or anti-CTLA-4 alone in a mouse model of melanoma [73]. Enhancer of zeste homolog 2 blockade led to reduced PD-L1 mRNA levels and a decrease in PD-L1+ Pax3+ in melanoma cells, which was maintained during concomitant IL-2cx or anti-CTLA-4 immunotherapy [74]. Taken together, these discoveries create a highly promising basis for combination studies using epigenetic and immune checkpoint therapy in patients with various cancers including liver cancer (Table 4).

**Table 4:** Ongoing clinical trials combining epigenetic drugs and immune checkpoint blockade therapy in cancers

Cancer type	Number	Immune checkpoint inhibitors	Epigenetic drugs	Stage	Status	Design	Trial NCT
HCC	90	Durvalumab	Guadecitabine	Phase 1	Recruiting	Single Group Assignment	NCT03257761
Unresectable NSCLC	41	Nivolumab and ipilimumab	ACY-241	Phase 1	Recruiting	Single Group Assignment	NCT02635061
Metastatic unresectable HER2-negative breast cancer	45	Pembrolizumab	Entinostat	Phase 1	Recruiting	Single Group Assignment	NCT02453620
Advanced solid tumors	30	Pembrolizumab	Entinostat	Phase 1	Recruiting	Randomized	NCT02909452
Unresectable stage III/IV melanoma	17	Ipilimumab	Panobinostat	Phase 1	Recruiting	Single Group Assignment	NCT02032810
Advanced CRC	30	Pembrolizumab	Romidepsin and/or 5-AZA	Phase 1	Recruiting	Randomized	NCT02512172
MSS advanced CRC	30	Pembrolizumab	Romidepsin and/or 5-AZA	Phase 1	Recruiting	Randomized	NCT02512172
MDS following DNMTi-failed therapy	27	Pembrolizumab	Entionstat	Phase 1	Recruiting	Single Group Assignment	NCT02936752
Advanced solid tumors or lymphomas	45	Nivolumab	RRx-001	Phase 1	Active, not recruiting	Single Group Assignment	NCT02518958
MM	19	Ipilimumab	SGI-110	Phase 1	Recruiting	Single Group Assignment	NCT02608437
MDS	73	Durvalumab with or without tremelimumab	Azacytidine	Phase 1	Recruiting	Non-Randomized	NCT02117219
Advanced cell carcinoma	62	Atezolizumab	Entinostat	Phase 1/2	Recruiting	Single Group Assignment	NCT03024437
Breast cancer	88	Atezolizumab	Entinostat	Phase 1/2	Recruiting	Randomized	NCT02708680
DLBCL	5	Rituximab	Belinostat	Phase 2	Active, not recruiting	Single Group Assignment	NCT01686165
Metastatic uveal melanoma	29	Pembrolizumab	Entinostat	Phase 2	Recruiting	Single Group Assignment	NCT02697630
DLBCL	42	Rituximab	Panobinostat	Phase 2	Active, not recruiting	Randomized	NCT01238692
Advanced solid tumors and NSCLC	119	Durvalumab	Mocetinostat	Phase 1/2	Recruiting	Single Group Assignment	NCT02805660
NSCLC and melanoma	202	Pembrolizumab	Entinostat	Phase 1/2	Recruiting	Non-Randomized	NCT02437136
HNSCC and SGC	49	Pembrolizumab	Vorinostat	Phase 1/2	Active, not recruiting	Single Group Assignment	NCT02538510
Stage IV NSCLC	100	Pembrolizumab	Vorinostat	Phase 1/2	Recruiting	Randomized	NCT02638090
DLBCL	83	Rituximab	Vorinostat	Phase 1/2	Active, not recruiting	Single Group Assignment	NCT00972478
Lymphoma/leukaemia	40	Rituximab	Vorinostat	Phase 1/2	Active, not recruiting	Single Group Assignment	NCT00918723
Advanced renal or urothelial cell carcinoma	42	Pembrolizumab	Vorinostat	Phase 2	Recruiting	Non-Randomized	NCT02619253
Hormone therapy-resistant breast cancer	87	Pembrolizumab	Vorinostat	Phase 2	Recruiting	Randomized	NCT02395627
AML	182	Nivolumab	5-AZA	Phase 2	Recruiting	Non-Randomized	NCT02397720
Metastatic CRC	31	Nivolumab	5-AZA	Phase 2	Active, not recruiting	Single Group Assignment	NCT02260440
Advanced/metastatic NSCLC	100	Nivolumab	5-AZA	Phase 2	Active, not recruiting	Randomized	NCT02546986
MDS	120	Nivolumab and/or ipilimumab	5-AZA	Phase 2	Recruiting	Non-Randomized	NCT02530463
Refractory/relapsed AML	37	Lirilumab	5-AZA	Phase 2	Active, not recruiting	Single Group Assignment	NCT02399917
MDS	12	Lirilumab and nivolumab	5-AZA	Phase 2	Active, not recruiting	Non-Randomized	NCT02599649
Metastatic melanoma	71	Pembrolizumab	5-AZA	Phase 2	Recruiting	Non-Randomized	NCT02816021
NSCLC	120	Nivolumab	5-AZA and/or entinostat	Phase 2	Recruiting	Randomized	NCT01928576
NSCLC	60	Nivolumab	5-AZA- CdR/tetrahydrouridine	Phase 2	Recruiting	Randomized	NCT02795923
Advanced solid tumors	60	Durvalumab	5-AZA	Phase 2	Recruiting	Single Group Assignment	NCT02811497
Advanced/metastatic NSCLC	100	Pembrolizumab	Oral azacytidine	Phase 2	Active, not recruiting	Randomized	NCT02546986
PR recurrent OC	38	Pembrolizumab	Guadecitabine	Phase 2	Recruiting	Single Group Assignment	NCT02901899
PR recurrent OC	20	Pembrolizumab	Oral azacytidine	Phase 2	Recruiting	Randomized	NCT02900560
MDS	120	Durvalumab	Oral azacytidine	Phase 2	Recruiting	Randomized	NCT02281084
MDS, AML	213	Durvalumab	Azacytidine	Phase 2	Active, not recruiting	Randomized	NCT02775903
Refractory/recurrent epithelial OC	138	Avelumab	Entinostat	Phase 2	Recruiting	Randomized	NCT02915523
DLBCL	304	Rituximab	5-AZA	Phase 3	Recruiting	Randomized	NCT02951156

*HCC* hepatocellular carcinoma, *NSCLC* Non-small cell lung cancer, *HER2* human epidermal growth factor receptor 2, *CRC* colorectal cancer, *5-AZA* Azacitydine, *MSS* Microsatellite stable, *MDS* Myelodysplastic syndromes, *DNMTi* DNA methyltransferase inhibitor, *MM* Multiple myeloma, *DLBCL* Diffuse large B cell lymphoma, *HNSCC* head and neck squamous cell carcinoma, *SGC* salivary gland cancer, *AML* Acute myeloid leukaemia, *OC* ovarian cancer

Combination therapy with immunotherapy and chemotherapy or radiation therapy are being studied and reported to be synergistic through multiple mechanisms. As more data of these combinations is available, it will likely improve outcomes for patients with this rare aggressive group of cancers, and we will also be able to develop further trials to upgrade our understanding of therapies targeting liver cancers. Therefore, immunotherapy offers hope to liver cancer patients with a dismal prognosis that has not seen significant changes in therapy for a long time.

## Limitations and perspectives of immune checkpoint therapy

Resistance to immune checkpoint blockades is still commonly observed in most cancer patients [75]. Failure of immune checkpoint inhibitors therapy can result from three categories: (1) mutations of the immunogenicity of cancer itself. The mutations influence expression of components of antigen-processing and presentation machinery (e.g., transporter associated with antigen processing, HLA class molecules, and β2 microglobulin), novel tumor-associated antigens (e.g., cancer-testis antigens, neoantigens), and cytokines; (2) expression of alternative immune checkpoint ligands on tumor cells (and/or immune cells). Expression of alternative co-inhibitory immune checkpoints (e.g., CTLA-4, TIM-3, LAG-3, and VISTA) has been associated with resistance to PD-1 blockade [76, 77]; or (3) defects in T cell infiltration. Diminished infiltration of T cells led to resistance to PD-1 blockade in melanoma patients [78]. However, epigenetic modifying agents including demethylating agents and histone deacetylase inhibitors may enable re-expression of immune related therapeutic genes, especially in combination of immunotherapy [79, 80]. They can also increase expression of immune checkpoints to synergize with immune checkpoint blockade therapy, leading to improving anti-tumor responses [81].

## Conclusions

Most liver cancers are diagnosed at an advanced stage, while the therapy is limited. Immune checkpoint therapy provides survival benefit for liver cancer treatment. Epigenetic regulation mechanistically and functionally links with immune checkpoints. Epigenetic mechanisms of checkpoint blocking prove to be promising in treating liver cancers and determining patient prognosis. Further investigations are required to explore the clinical potential in combination with epigenetic and immune checkpoint therapy for liver cancer treatment.

## Abbreviations

APC: Antigen presenting cell
ASCO: American Society of Clinical Oncology
BTLA: B- and T-lymphocyte attenuator
CTLA-4: Cytotoxic T lymphocyte–associated antigen 4
DNMT1: DNA methyltransferase 1
DNMTi: DNA methyltransferase inhibitors
GITR: Glucocorticoid-induced tumor necrosis factor receptor-related gene
HBV: Hepatitis B virus
HCC: Hepatocellular carcinoma
HDACi: Histone deacetylase inhibitors
HLA: Human leukocyte antigens
HVEM: Herpesvirus entry mediator
ICC: Intrahepatic cholangiocarcinoma
IDO: Indoleamine 2,3-dioxygenase
KIRs: Killer cell immunoglobulin-like receptors
LAG-3: Anti-lymphocyte activation gene-3
lncRNAs: long noncoding RNAs
miRNAs: microRNAs
PD-1: Programmed cell death protein-1
PD-L1: Programmed cell death ligand 1
TAA: Tumor-associated antigens
TAM: Tumor-associated macrophages
TILs: Tumor-infiltrating lymphocytes
Tim-3: T-cell immunoglobulin- and mucin-domain-containing molecule-3
Tregs: Regulatory T cells
VISTA: V-domain Ig suppressor of T-cell activation
